# Antigen-specific CD4^+^ T cells exhibit distinct transcriptional phenotypes in the lymph node and blood following vaccination in humans

**DOI:** 10.21203/rs.3.rs-3304466/v1

**Published:** 2023-09-15

**Authors:** Philip Mudd, Nicholas Borcherding, Wooseob Kim, Michael Quinn, Fangjie Han, Julian Zhou, Alexandria Sturtz, Aaron Schmitz, Tingting Lei, Stefan Schattgen, Michael Klebert, Teresa Suessen, William Middleton, Charles Goss, Chang Liu, Jeremy Crawford, Paul Thomas, Sharlene Teefey, Rachel Presti, Jane O’Halloran, Jackson Turner, Ali Ellebedy

**Affiliations:** Washington University School of Medicine; Washington University; Korea University College of Medicine; Washington University School of Medicine; Washington University School of Medicine; Washington University in St Louis; Washington University School of Medicine; Washington University School of Medicine; Washington University School of Medicine; St Jude Children’s Research Hospital; Washington University School of Medicine; Washington University School of Medicine; Washington University School of Medicine; Division of Biostatistics, Washington University in St.Louis; Washington University School of Medicine; St Jude Children’s Research Hospital; St. Jude Children’s Research Hospital; Washington University School of Medicine; Washington University School of Medicine; Department of Emergency Medicine, Washington University in St.Louis; Washington University School of Medicine; Washington University School of Medicine

**Keywords:** CD4+ T cell, T follicular helper cell, lymph node, SARS-CoV-2, mRNA vaccination

## Abstract

SARS-CoV-2 infection and mRNA vaccination induce robust CD4^+^ T cell responses that are critical for the development of protective immunity. Here, we evaluated spike-specific CD4^+^ T cells in the blood and draining lymph node (dLN) of human subjects following BNT162b2 mRNA vaccination using single-cell transcriptomics. We analyze multiple spike-specific CD4^+^ T cell clonotypes, including novel clonotypes we define here using Trex, a new deep learning-based reverse epitope mapping method integrating single-cell T cell receptor (TCR) sequencing and transcriptomics to predict antigen-specificity. Human dLN spike-specific T follicular helper cells (T_FH_) exhibited distinct phenotypes, including germinal center (GC)-T_FH_ and IL-10^+^ T_FH_, that varied over time during the GC response. Paired TCR clonotype analysis revealed tissue-specific segregation of circulating and dLN clonotypes, despite numerous spike-specific clonotypes in each compartment. Analysis of a separate SARS-CoV-2 infection cohort revealed circulating spike-specific CD4^+^ T cell profiles distinct from those found following BNT162b2 vaccination. Our findings provide an atlas of human antigen-specific CD4^+^ T cell transcriptional phenotypes in the dLN and blood following vaccination or infection.

## Introduction

The SARS-CoV-2 pandemic provided a unique opportunity to study primary human immune responses to a novel pathogen and the immunodominant spike antigen from that pathogen incorporated into various highly-effective vaccine platforms. Messenger RNA (mRNA) vaccines, approved for use in humans by worldwide regulatory bodies for the first time during the COVID-19 pandemic, engender some of the strongest immune responses to the SARS-CoV-2 spike antigen. These strong responses include high-frequency circulating spike-specific CD4^+^ T cells^1,2^ and spike-specific T follicular helper cells (T_FH_) in the draining lymph node (dLN)^3^.

T_FH_ are CD4^+^ T cells that support the development and maintenance of germinal center (GC) B cells in secondary lymphoid organs^4,5^. T_FH_ function by providing appropriate co-stimulation and cytokine survival signals to B cells in GCs throughout the process of antibody class switch, affinity maturation, long-lived plasma cell development, and memory B cell development^4–6^. In murine models, functional T_FH_ are absolutely required for productive GC and the development of both memory B cells and long-lived plasma cells^7–11^.

Due to limitations in sampling human secondary lymphoid organs, the phenotype of human T_FH_ in the dLN following antigen challenge is just beginning to be explored^3,12^. To date, human immunologists have studied GC and T_FH_ responses in easily accessible tissue compartments, including blood and discarded clinical tonsillectomy tissue^13–19^. Evaluations of human lymph node tissue biopsies or these tissues at autopsy have yielded insights into the phenotype of human lymph node T_FH_^20–23^. However, these studies have been limited to the exploration of T_FH_ phenotypes in a steady state. The study of human antigen-specific T_FH_ in secondary lymphoid organs following acute infection or vaccination is even more limited^3,15,24^ and rarely includes study of antigen-specific responses at the single-cell level.

We recently established a system to probe human GC responses in the axillary dLN following deltoid intramuscular vaccination using serial fine needle aspiration (FNA) of ultrasound-localized dLN^25,26^. At the beginning of the COVID-19 vaccine rollout, we initiated studies of a human cohort receiving SARS-CoV-2 mRNA vaccines using this model system^3,26^. We previously showed strong induction of spike-specific T_FH_ responses in the dLN in this cohort^3^. In the present study, we performed single-cell RNA sequencing (RNA-seq) to obtain complete transcriptomes with matched T cell receptor (TCR) sequencing from more than 200,000 T cells found in blood and dLN of six SARS-CoV-2-naïve, HLA-DPB1*04^+^ human subjects 7 to 180 days after vaccination with the second dose of a primary two dose BNT162b2 mRNA vaccine series. Our resulting data provide an atlas of total and antigen-specific T_FH_ transcriptional phenotypes in the human dLN during an ongoing GC. Using a novel reverse epitope discovery technique we developed to integrate biochemical properties of TCR complementarity determining region 3 (CDR3) amino acids and transcriptional profiles in single cells to predict antigen-specificity, we expand the number of known antigen-specific TCR in our dataset, confirm these paired TCR are spike-specific, and analyze the transcriptional dynamics of multiple lineages of antigen-specific CD4^+^ T cells in the blood and dLN following vaccination. Finally, we incorporate analysis of antigen-specific CD4^+^ T cells found in blood from a cohort of HLA-DPB1*04^+^ human subjects following primary infection with SARS-CoV-2 and compare these responses to spike-specific memory CD4^+^ T cells found after vaccination.

## Results

### Diverse T cell transcriptional landscape in the blood and dLN post-vaccination

**Figure 1:**
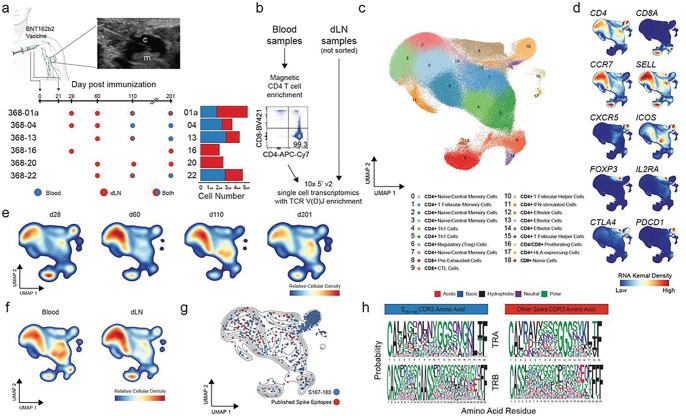
Single-cell T cell landscape of dLN and blood following SARS-CoV-2 mRNA vaccination. **a.** Experimental design. Schematic of sample collection with fine-needle aspiration, experimental time points, tissue(s) collected, and a summary of overall cell numbers by donor and tissue. For each sample collection, a technical replicate was performed and sequenced. **b.** Experimental design and representative flow cytometry following magnetic T cell enrichment. **c.** UMAP of 219,283 dLN and blood T cells that passed quality control filtering and contained a paired TCR sequence. Cluster annotation based on canonical subtype markers and automated annotation via SingleR and ProjecTIL. **d.** Gene-weighted density estimates overlaid on the UMAP coordinates for general T cell markers. **e.** Relative density of cells by collection time point. **f.** Relative density of cells by tissue. **g.** Localization of previously identified spike-specific TCRs. TCRs that bind S_167–180_ are highlighted in blue and other spike-specific TCRs are in red. **h.** Aligned TRA and TRB CDR3 motifs for S_167–180_ reactive TCRs and other spike-specific TCRs.

We leveraged the high prevalence of spike-specific CD4^+^ T cells recognizing the immunodominant S_167 – 180_ epitope^3^ in SARS-CoV-2 mRNA-vaccinated HLA-DPB1*04^+^ individuals in order to evaluate transcriptional phenotypes at the antigen-specific level over time in blood and dLN CD4^+^ T cells. We performed single-cell RNA-seq and paired TCR sequencing of HLA-DPB1*04^+^ subjects who participated in a prospective observational cohort study following a two-dose BNT162b2 primary vaccine series^3,27^ Demographics and HLA-typing of the clinical cohorts are recorded in **Suppl. Table 1** and **Suppl. Table 2.** We prepared and sequenced total dLN cells from fine-needle aspiration samples and magnetically enriched total CD4^+^ cells from temporally matched blood samples obtained from four of the six subjects (Fig. 1a & 1b). All subjects had an ongoing spike-specific GC B cell response in the evaluated dLN at each time point^27^.

Our data set included 23 samples of distinct tissues/time points post-vaccination (Fig. 1a). We sequenced a total of 219,283 individual T cells which passed all transcriptional quality metrics and contained a paired TCR sequence (Fig. 1c). The lack of CD4^+^ selection during dLN sample preparation and small amounts of CD8^+^ T cell contamination from the magnetic separation step for blood samples (Fig. 1b) resulted in CD8^+^ T cells being included in the data set. We identified 18 transcriptional T cell clusters based upon Uniform Manifold Approximation and Projection (UMAP) analysis (Fig. 1c). Following annotation using granular cell types with canonical markers and reference atlases (**Suppl. Figure 1**), we identified two T_FH_ subsets (C10 and C15) and one memory T_FH_ subset (C1) that co-localized in the same region of the UMAP. Common T cell markers clearly separated in the UMAP projection (Fig. 1d). All transcriptional clusters were present at each separate time point (Fig. 1e) and in both the PBMC and dLN tissues (Fig. 1f).

We found individual T cells with published^3,28,29^ SARS-CoV-2-specific TCR CDR3 sequences, including the immunodominant HLA-DPB1*04-restricted S_167 – 180_ CD4^+^ T cell epitope^3^, throughout the UMAP projection (Fig. 1g). S_167 – 180_-specific TCR were primarily localized in the T_FH_ clusters, consistent with the enrichment of our data set for dLN tissue sampled from a cohort of HLA-DPB1*04^+^ individuals during an ongoing GC response. Alignment of previously published spike-specific TCR alpha chain (TRA) and TCR beta chain (TRB) CDR3 sequences contained in our dataset revealed overlaps in the predominant biochemical signature of each amino acid residue (Fig. 1h).

### Transcriptional phenotypes of T_FH_ in the dLN following vaccination

**Figure 2:**
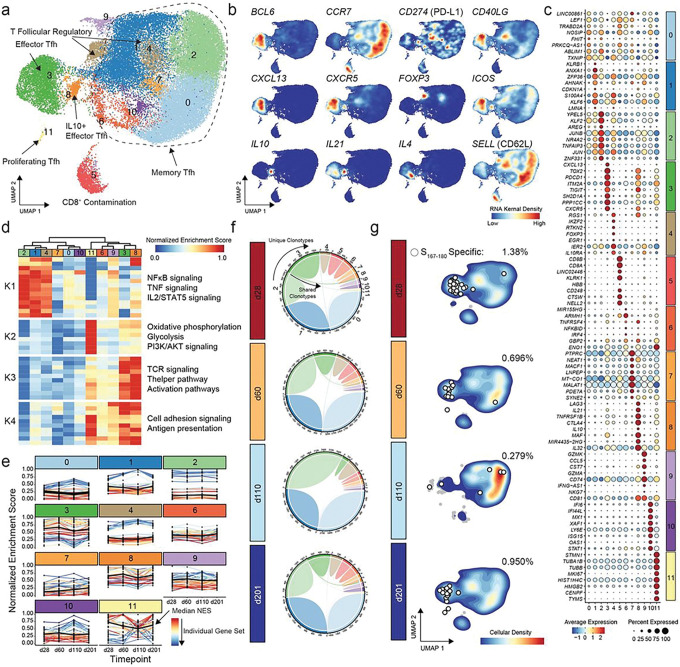
Transcriptional phenotypes of dLN T_FH_ following SARS-CoV-2 mRNA vaccination. **a.** UMAP of T_FH_ from the dLN of all donors at all time points. **b.** Gene-weighted density estimates of indicated transcripts overlaid on the UMAP. **c.** Top 8 or fewer cluster-defining differentially expressed genes. Dot size represents the percentage of cells expressing the gene, and color is assigned based on scaled expression value. **d.** Median gene set enrichment results for the significantly altered gene sets by cluster with each K indicating a separate k-means cluster gene set **e.** Normalized gene set enrichment values by cluster and time point. Colors indicate individual gene sets. **f.** Circos plot by time point post-vaccination showing overlap of unique individual TCR clonotypes with ribbons between clusters representing overlapping clonotypes. **g.** Relative density of cells by time point with S_167–180_ -specific TCR represented by white dots.

To analyze the phenotypic dynamics of human T_FH_ in the dLN following vaccination, we selected all dLN T cells found in the T_FH_ and T_FH_ memory clusters C1, C10, or C15 in the total dataset from Fig. 1c and generated a new UMAP of this subset of T cells. We found 12 distinct phenotypic clusters (denoted c0 through c11) of human dLN T_FH_ (Fig. 2a). These were principally classified as effector T_FH_ (c3, c6, c8, and c9), proliferating T_FH_ (c11), regulatory T_FH_ (c4) and memory T_FH_ (c0, c1, c2, c7, and c10). A single distinct cluster (c5) represented CD8^+^ T cells found in the dLN and was not considered further.

Classically defined germinal center T_FH_ (GC T_FH_)^4^, distinguished by very high expression of *CXCR5, PDCD1, BCL6,* and *CXCL13*, composed the largest effector T_FH_ cluster (c3, Fig. 2a–c). A second large effector T_FH_ cluster consisted of the previously described^20,21^ IL-10-expressing T_FH_ subset (IL-10^+^ T_FH_, c8, Fig. 2a–c). These two effector T_FH_ clusters shared many characteristics, including the highest expression of canonical GC T_FH_ markers, *CXCR5, PDCD1,* and *BCL6*, and clustered together in hierarchical clustering analysis of gene sets (Fig. 2d). They shared the highest expression levels of genes related to TCR signaling, T helper pathways, activation pathways, cell adhesion signaling and antigen presentation in gene set enrichment analysis (Fig. 2d). They also shared high expression of genes related to increased metabolic activity with elevated expression levels of genes involved in oxidative phosphorylation, glycolysis and PI3K/AKT signaling (Fig. 2d). Both subsets maintained relatively consistent expression of distinguishing gene sets over time throughout the duration of the GC (Fig. 2e) and shared the largest number of identical paired TCR clonotypes among all dLN T_FH_ clusters suggesting significant overlap in clonal populations recruited to both effector subsets (Fig. 2f). Despite the close relationship between these two subsets of human dLN effector T_FH_, they exhibited clear differences in cytokine gene expression with exclusive *IL10* expression and much higher *IL21* expression in IL-10^+^ T_FH_ and much higher *IL4* expression in GC T_FH_. IL-10^+^ T_FH_ exhibited high *CTLA4* expression, but lacked *FOXP3* expression (Fig. 2b and 2c), as previously described^20^.

Two additional effector T_FH_ clusters, c6 and c9, exhibited much lower expression of the classical GC T_FH_ markers *CXCR5, PDCD1* and *BCL6*, but shared a related gene set expression profile that segregated with GC T_FH_ and IL-10^+^ T_FH_ in hierarchical clustering analysis (Fig. 2d). The functional significance of these two T_FH_ populations is not clear, but genes distinguishing these T_FH_ from other subsets (Fig. 2c) suggest interesting immunologic roles. c6 cells exhibited high expression of the microRNA *MIR155HG*, a transcript associated with increased inflammation through regulation of *SOCS1* and many other genes^30^ that has also recently been shown to encode a short functional peptide, miPEP155, that modulates class II antigen presentation^31^. This subset expressed the highest level of the T cell transcriptional regulators *IRF4* and *NFKBID*, suggesting a transitional phenotype in cells that may ultimately develop into other CD4^+^ T cell subsets. c6 cells exhibited TCR clonotypic overlap during the ongoing GC reaction with several other T_FH_ subsets including c1, c2, c4, c7 and IL-10^+^ T_FH_ (Fig. 2f), but had the highest frequency of clonotypic overlap with cells found in the GC T_FH_, demonstrating an enduring relationship between these two subsets over time. Cells belonging to the other non-GC T_FH_ subset of effector T_FH_, c9, uniquely expressed high levels of cytotoxic genes, including granzyme A (*GZMA*), granzyme K (*GZMK*), and natural killer cell granule protein 7 (*NKG7*). These cells exhibited clonal overlap with the memory T_FH_ cells found in c1 at all time points evaluated and had minimal clonal overlap with other subsets (Fig. 2f), suggesting a unique lineage distinct from GC T_FH_. It is intriguing to speculate that this subset may be related to previously identified “cytotoxic” T_FH_ characterized elsewhere^15^.

Proliferating T_FH_, found in c11, expressed the proliferation marker *MKI67* (Fig. 2c) and exhibited specific gene expression patterns and gene set enrichment characteristics that aligned these cells with effector T_FH_ (Fig. 2d). While a spatiotemporal relationship between the proliferating T_FH_ found in c11 and other effector T_FH_ cannot be established from these data alone, it is notable that unique paired TCR clonotypes were shared specifically between c11 and the c3/c8 clusters at each individual time point evaluated (Fig. 2f). This demonstrates a close and ongoing relationship between the GC T_FH_ / IL-10^+^ T_FH_ and proliferating T cells of matched clonotype throughout the course of the human GC rather than a burst of proliferation early that is simply maintained over time.

Memory T_FH_ (c0, c1, c2, c7, and c10) populations found in the dLN were typified by the lower relative expression of genes involved in oxidative and glycolytic metabolism pathways, genes involved in TCR signaling and genes involved in cell adhesion signaling (Fig. 2d). Cluster-defining genes in these subsets included transcription factors involved in maintaining long-term T cell responsiveness and homeostasis such as *KLF2*^32,33^, *JUN*^34^, *JUNB*^35^, and *KLF6*^33^. The c0, c2 and c10 memory T_FH_ clusters demonstrated very few TCR clonal overlaps with other T_FH_ subsets (Fig. 2f). c7 memory T_FH_ cells exhibited clonal overlap with GC T_FH_ cells at every evaluated time point. c1 memory T_FH_ cells also displayed a relatively large degree of clonal overlap with GC T_FH_ cells, but also showed overlap with regulatory T_FH_ cells, IL-10^+^ T_FH_ cells, c9 effector T_FH_ cells, and c7 memory T_FH_ cells. These findings suggest a close relationship between GC T_FH_ and the memory T_FH_ populations found in c7 and c1.

TCR sequences previously established to be specific for the S_167 – 180_-epitope^3^ were found primarily in GC T_FH_ and IL-10^+^ T_FH_ during the ongoing GC reaction (Fig. 2g). S_167 – 180_-specific cells maintained a relatively consistent frequency (approximately 0.8%) as a percentage of total T_FH_ cells overtime during the GC (Fig. 2g). We did, however, find small numbers of S_167 – 180_-specific T_FH_ in proliferating T_FH_ (c11) and memory T_FH_ (c2, c0, and c1) clusters during the experimental time course.

### Development of Trex to identify additional populations of antigen-specific CD4^+^ T_FH_

**Figure 3:**
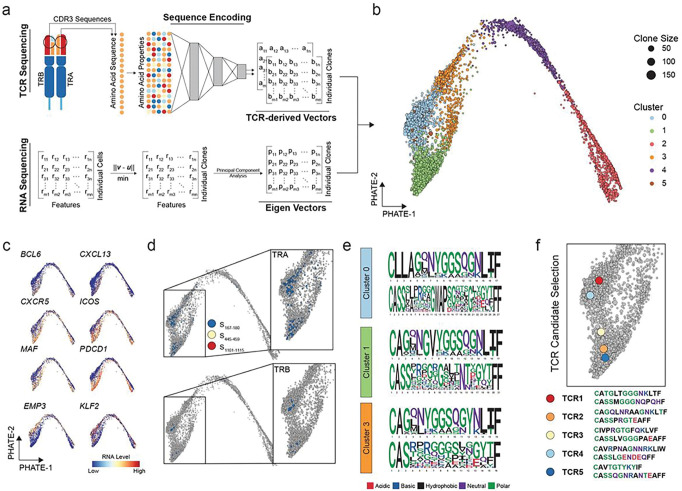
Coembedding of single-cell TCR and RNA values from dLN T_FH_ using Trex. **a.** Graphical representation of computational embedding using TCR CDR3 amino acid sequences and single-cell RNA levels. The resulting three matrices are rescaled based on nearest neighbors and the corrected values are then used for dimensional reduction. **b.** PHATE projection of the tri-modal (RNA, TRA, and TRB) embedding of dLN T_FH_ by clonotype with size of the clonotype indicated by dot size. **c.** Representative RNA expression of selected genes overlaid onto the PHATE projection. **d.** Localization of previously established spike-specific TRA (upper panel) and TRB (lower panel). **e.** Aligned TRA and TRB motifs for given PHATE clusters with spike-specific TCRs. **f.** Location and sequence for the five selected spike-specific candidate TCR clonotypes derived from Clusters 0 and 1 that have a TCR chain appearing in > 1 donor and have not been previously described as spike-specific.

Previously reported TCR sequencing in a more limited context in this model system suggested additional dominant and subdominant populations of likely antigen-specific T_FH_^3^. To expand the number of known SARS-CoV-2 spike-specific TCR sequences in the present dataset, we developed a new method to identify antigen-specific CD4^+^ T cells that integrates the biochemical properties of amino acids found in the TCR sequence and transcriptional signatures of specific cells (Fig. 3). We term this novel methodology Trex (**T** cell **R**eceptor and **Ex**pression). Trex combines the TCR sequence and transcriptional signature using a co-embedding approach of the RNA transcriptome and the latent dimensional embeddings of both the TRA and TRB CDR3 sequences for each clonotype (Fig. 3a, “TCR-derived vectors”). Model hyperparameters were empirically based using a bootstrap approach (**Suppl. Figure 2a and 2b**). Each model in Trex demonstrated high fidelity in the return of unique latent dimensional embeddings across sequences (**Suppl. Figure 2c**) and runtimes less than 20 seconds for 50,000 unique TCR sequences (**Suppl. Figure 2d**). The latent dimensional embeddings are based on the output of neural network-based transformers, called variational autoencoders, which transform the amino acid sequence of each clonotype into a matrix based on Kidera factors before encoding (Fig. 3a). For a given clonotype, a centroid-like approach is used to select a best representative cell to use for RNA expression based on the minimal Euclidean distance across the calculated principal components (Fig. 3a), similar to the previously described method, clonotype neighbor graph analysis (CoNGA)^36^. For a given clonotype, the TRA, TRB, and RNA vectors are then co-embedded, and a nonlinear dimensional reduction is calculated to represent an immune response at both the transcriptional and repertoire levels simultaneously.

We used Trex to examine all dLN T_FH_ cells included in Fig. 2a, and generated a PHATE-based manifold of the resulting data (Fig. 3b). Transcription of various T_FH_ genes partitioned throughout the manifold (Fig. 3c), consistent with the inclusion of both transcriptional and TRA/TRB properties in the model. We found that previously known spike-specific TCR clonotypes co-localized into unique and very focal areas within clusters 0, 1, and 3 of the PHATE-based manifold (Fig. 3d). TRA and TRB CDR3 in these three clusters shared related amino acid biochemical properties (Fig. 3e) similar to that observed with published spike-specific clonotypes (Fig. 1h). We compared the results of the Trex PHATE-based manifold to those obtained using CoNGA (**Suppl. Figure 3a and 3b**). When comparing the overlap of the nearest neighbors between the Trex- and CoNGA-derived TCR vectors, we found overlap in the neighbors called for a subset of spike-specific clones, but not across all clones (**Suppl. Figure 3c**). CoNGA TCR-based clustering centralized spike-specific clones into a single cluster, whereas Trex-based clusters exhibited multiple small spike-specific-predominant clusters (**Suppl. Figure 3d and 3e**).

We hypothesized that clonally-expanded dLN T_FH_ with at least one public TRA or TRB shared in two or more subjects located within Cluster 0, 1, or 3 in close proximity to other known spike-specific CD4^+^ T cell clonotypes would have a high probability of being spike-specific. We chose five TCR candidates that fit these criteria to test this hypothesis (Fig. 3f). The five candidates were distributed uniquely into multiple CoNGA TCR-based clusters and Trex-based clusters (**Suppl. Figure 3f**). We synthesized these five TCR and cloned them into a retroviral transduction system^37^ and transduced primary human CD4^+^ T cells with each TCR construct. We mapped the responsiveness of each TCR transductant to overlapping spike peptides *in vitro* to determine epitope specificity (**Suppl. Figure 4**). All five candidate TCR were spike-specific. Interestingly, the TCR 2 transductant line mapped to S_167 – 180_ and bound the HLA-DPB1*04:01-S_167 – 180_ tetramer (**Suppl. Figure 4**), but did not share the TRA motif we previously characterized as S_167 – 180_-specific^3^ and therefore was not included in our initial analysis of this epitope in the dataset. We next selected all members of the TRA/TRB families with highly-related TCR to the index TCR candidates that we experimentally determined were spike-specific. Using this methodology, we added 74 spike-specific T cells to our dLN T_FH_ dataset and expanded the total number of spike-specific dLN T_FH_ cells from 164 to 238 (**Suppl. Table 3**).

### Antigen-specific T_FH_ transcriptional profiles vary over time during an ongoing GC

**Figure 4:**
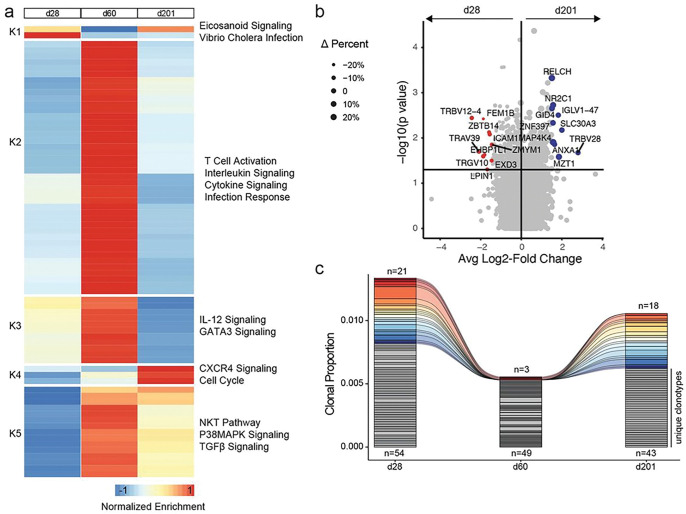
Antigen-specific T_FH_ dynamics in the dLN. **a.** Median gene set enrichment results for immune-related gene sets by time point. **b.**Volcano plot of differential gene expression comparing spike-specific T_FH_ at d28 (n=98, red) and d201 (n=73, blue). Size is based on the difference in the percentage of cells expressing the gene at d28 compared to d201. **c.** Clonal proportion of spike-specific dLN T_FH_ repertoire at indicated time points. The top number on the bar plot indicates the number of clones at the given time point shared across time points, whereas the number on the bottom indicates spike-specific clones unique to the time point.

Using our expanded dataset (**Suppl. Table 3**), we next explored the phenotypic dynamics of spike-specific T_FH_ in the dLN during an ongoing GC response (Fig. 4). All six BNT162b2 vaccinees included in this study demonstrated ongoing spike-specific GC B cell responses in the dLN at all time points^26,27^. The dLN T_FH_ from d110 included significantly fewer spike-specific cells (n = 12) when compared with the d28, d60, and d201 time points, therefore we excluded d110 from this analysis.

Gene set enrichment analysis revealed significant differences in T cell activation, interleukin signaling, cytokine signaling and infection response genes over time with significant enrichment of these pathways in antigen-specific T_FH_ found at the peak of the GC response on d60 (Fig. 4a). IL-12 signaling, GATA3 signaling, NKT pathway genes, P38MAPK signaling and TGF beta signaling pathways were also upregulated at d60. Gene sets representing CXCR4 signaling and cell cycle progression were significantly enriched in antigen-specific T_FH_ at the end of the GC response (d201, Fig. 4a).

Despite relatively similar gene expression at the beginning (d28) and the end (d201) of the ongoing GC (Fig. 4a), we did detect several differentially expressed genes (Fig. 4b, **Suppl. Table 4**). These included TCR genes, consistent with clonal differences in T cells found at the two time points. However, we also detected several differentially expressed coding transcripts. Early antigen-specific dLN T_FH_ (d28) exhibited higher expression of *ICAM1*, suggesting enhanced activation and clustering of T cells during the early stage of the human antigen-specific GC. *ZBTB14*, a poorly-characterized member of the zinc finger and BTB domain family of transcription factors that includes Bcl-6 (*ZBTB27*)^38^, was upregulated in antigen-specific T_FH_ early in the GC. Genes involved in cholesterol metabolism (*RELCH*), ubiquitination (*GID4*), and intracellular signaling (*MAP4K4, ANXA1*) were differentially regulated in antigen-specific T_FH_ late in the GC.

We next evaluated paired TCR clonotypes found in antigen-specific T_FH_ cell populations in the dLN at various time points (Fig. 4c). We tracked several identical paired TCR clonotypes observed at more than one time point during the ongoing GC (colored clonotypes, Fig. 4c). These accounted for between 5% and 28% of the total number of cells sequenced in the dataset, demonstrating persistence or proliferation of clonally-identical antigen-specific T_FH_ within the GC over time.

### Minimal paired TCR sequence overlap between circulating and dLN CD4^+^ T cells

After tracking several clonally-identical antigen-specific T_FH_ in the dLN over time and showing that these cells make up a significant fraction of antigen-specific T_FH_ in the dLN (Fig. 4c), we next sought to determine if clonally-identical populations of antigen-specific CD4^+^ T cells could be found in the blood during the ongoing GC reaction. We first assessed our dataset for identical paired TRA/TRB sequences in all sequenced T cells in the dLN and blood from three of the six members of the cohort with temporally matched blood and dLN samples obtained at d110. We included a paired blood and dLN sample from subject 368–01a at d201. This analysis included all sequenced T cells, including CD4^+^ and CD8^+^ T cells. To our surprise, despite substantial numbers of spike-specific CD4^+^ TCR clonotypes identified in these individuals in both dLN and blood compartments at these time points, we found no clonally-identical paired TCRs present in both the blood and dLN at these four matched time points during the ongoing GC reaction (data not shown).

**Figure 5:**
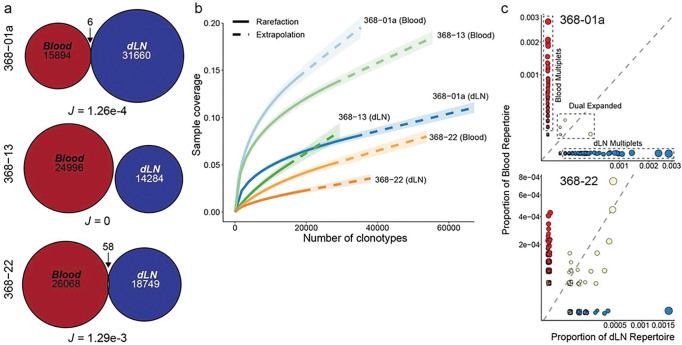
Minimal overlap in clonal TCR repertoire between dLN and blood. **a.** Clonal overlap across all time points for blood and lymph nodes in indicated patients with the Jaccard stability index calculated. **b.** Sample-size-based rarefication and extrapolation for clonotypes of all time points for donors with paired blood and dLN samples. **c.** Scatter plot showing the localization and categorization of TCR clonotypes found in the two subjects with overlapping clonotypes.

Expanding this analysis beyond our initial search for matched paired TCR at specific time points, we found minimal overlap between the paired TCR repertoire in the blood and dLN when all blood and dLN samples obtained at each available time point from these three individuals were analyzed together (Fig. 5a). Indeed, we found only 6 overlapping TCR out of 47,560 sequenced in subject 368–01a, no overlapping TCR out of 39,280 sequenced in subject 368 – 13, and 58 overlapping TCR out of 44,817 sequenced in subject 368 – 22 (Fig. 5a). Rarefaction analysis suggested adequate sampling depth to fully represent the diversity of the TCR repertoire in both the dLN and blood compartments in these individuals (Fig. 5b), implying that sequencing depth was not the primary limiting factor in the lack of significant clonal overlap and that these two compartments represent relatively unique populations of clonally diverse T cells.

The majority of shared clonotypes between blood and dLN identified in subjects 368–01a and 368 – 22 represented relatively infrequent paired TCR clonotype populations found in only one or two cells in either the blood or the dLN (Fig. 5c, **Suppl. Tables 5 and 6**) rather than clonally expanded populations. There were four notable exceptions - all of which were CD8^+^ T cell populations: one found in 29 T cells from subject 368 – 22 (20 in blood, 9 in dLN), a second found in 21 T cells from subject 368 – 22 (12 in blood, 9 in dLN), a third found in 14 T cells from subject 368 – 22 (6 in blood, 8 in dLN), and a fourth found in 13 T cells from subject 368–01a (1 in blood, 12 in dLN). Almost half (48%) of the populations with overlapping TCR clonotypes were CD8^+^ T cells based upon transcriptional profile. This is especially striking due to the magnetic enrichment of the blood samples for CD4^+^ T cells to > 97% purity prior to sequencing (Fig. 1b), suggesting that the frequency of overlapping blood and dLN clonal T cell populations for CD4^+^ T cells is substantially less than that observed for CD8^+^ T cells. None of the 58 overlapping TCR clonotypes in subject 368 – 22 contained CDR3 sequences of known SARS-CoV-2 spike-specific CD4^+^ T cells, including those determined to be spike-specific in this manuscript (**Suppl. Table 6**). Three of the six overlapping clonotypes in subject 368–01a were SARS-CoV-2 spike-specific CD4^+^ T cells (**Suppl. Table 5**), two were S_167 – 180_-specific and a third was S_120 – 136_-specific (TCR5 from Fig. 3f). None of these three spike-specific CD4^+^ T cells were found in matched blood and dLN samples obtained from the same time point (**Suppl. Table 5**). All three overlapping clonotypes were found relatively early (d28, d60) in the dLN and late (d110, d201) in the blood, perhaps indicating the emergence of memory T_FH_ from the dLN to the circulation late in the course of the GC.

We hypothesized that the significant number of overlapping clonotypes that are not known spike-specific clonotypes may be from clonal T cell populations over-represented due to the enlarged nature of the human memory T cell repertoire for several commonly encountered antigens such as CMV and EBV. We analyzed the overlapping TCR sequences against EBV- and CMV-specific CDR3 published on the Immune Epitope Database and Analysis Resource website (iedb.org). Consistent with this hypothesis, at least one chain of 33% (2 of 6) and 16% (9 of 58) of overlapping TCR clonotypes found in subjects 368–01a and 368 – 22, respectively, were previously classified as EBV- or CMV-specific (**Suppl. Tables 5 and 6**).

### SARS-CoV-2-specific CD4^+^ T cells maintain distinct transcriptional signatures in the blood and the dLN

**Figure 6:**
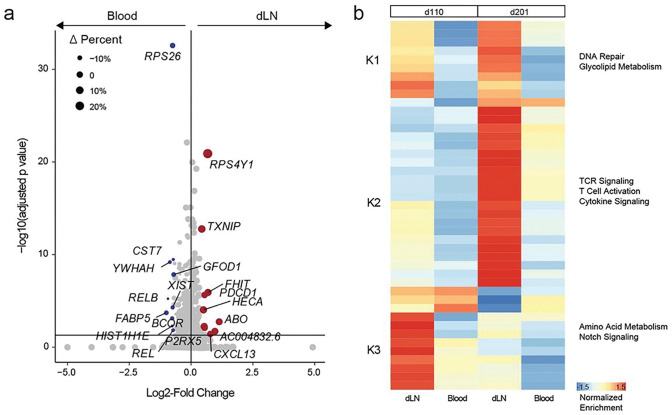
Antigen-specific T cell dynamics in the blood and dLN. **a.** Volcano plot of differential gene expression comparing spike-specific T cells from lymph node (n=533, red) with blood (n=938, blue). Size is based on the difference in the percentage of cells expressing the gene in dLN compared to blood. **b.** Z-scaled median gene set enrichment results for immune-related gene sets by tissue and time point.

We next explored the transcriptional signatures of SARS-CoV-2 spike-specific CD4^+^ T cells found in blood and dLN samples collected at d110 and d201 (Fig. 6). We included both T_FH_ and non-T_FH_ CD4^+^ T cells from all sequenced dLN and blood samples from these time points in the cohort (Fig. 1a) with TCR that were S_167 – 180_-specific, those with previously published spike-specific TCR, and the five new clonotype families discovered using Trex. A broad evaluation of transcriptional differences between the blood and dLN compartments at these two time-points revealed several significant transcriptional differences (Fig. 6a and **Suppl. Table 7**). These included upregulation of *PDCD1* and *CXCL13* in dLN antigen-specific CD4^+^ T cells, two genes important in T_FH_ biology^39,40^. *REL* and *RELB*, genes involved in canonical and non-canonical NF-*κ*B signaling respectively, were significantly upregulated in blood antigen-specific CD4^+^ T cells compared with dLN antigen-specific CD4^+^ T cells. The CST7 gene, which encodes a cysteine protease inhibitor, was also significantly upregulated in blood antigen-specific CD4^+^ T cells.

Gene set enrichment analysis of these data revealed substantial similarity between the two antigen-specific peripheral blood time points and some differences between the two dLN antigen-specific CD4^+^ transcriptional profiles (Fig. 6b). Antigen-specific CD4^+^ T cells in the two blood samples had less DNA repair and Glycolipid metabolism signaling than the dLN samples. We observed enrichment of TCR signaling, T cell activation and Cytokine signaling pathways in the d201 dLN samples. The d110 dLN samples had significantly elevated amino acid metabolism and Notch signaling when compared with the other samples.

### Antigen-specific circulating blood CD4^+^ T cells induced by infection are transcriptionally distinct from those induced by mRNA vaccination

**Figure 7:**
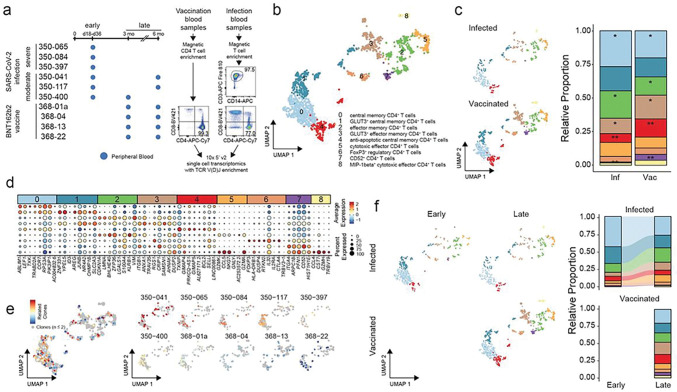
Comparison of circulating blood antigen-specific CD4^+^ T cells induced by infection or mRNA vaccination. **a.** Schematic, timeline and representative purity of peripheral blood samples from infected (n=6) and vaccinated (n=4) subjects. **b.** UMAP projection of all antigen-specific CD4^+^ T cells in 10 subjects, including 1,497 total cells: 693 from infected and 804 from vaccinated. **c.** UMAP projection and proportion breakdown of cluster composition for samples collected at 3–6 months in infected (n=289) and vaccinated (n=804) subjects. * corrected p-value < 0.05; ** corrected p-value < 0.01 **d.** Top 8 or fewer cluster-defining differentially expressed genes. **e.** TCR cluster assignments based on normalized Levenshtein distance of the CDR3 sequence across subject samples. Only cluster assignments with more than two clonotypes were retained. **f.** UMAP and proportion breakdown of antigen-specific CD4^+^ T cells in infected subjects at early and late time points. Vaccinated subjects at late time point are included as a reference.

Leveraging our ability to detect large numbers of spike-specific CD4^+^ T cells in HLA-DPB1*04^+^ individuals, we next compared the transcriptional phenotype of circulating peripheral blood antigen-specific CD4^+^ T cells in human subjects following BNT162b2 mRNA vaccination to the phenotype found in cells obtained from individuals following acute symptomatic primary SARS-CoV-2 infection. To do this, we generated a new dataset that included single cell RNA-seq of PBMC collected at the d110 and d201 time points from four mRNA vaccinated individuals (Fig. 1a), and included blood PBMC samples from six DPB1*04^+^ individuals hospitalized with moderate or severe COVID-19 in the St. Louis area during the first wave of the pandemic during the spring and summer of 2020, prior to the introduction of vaccines^41^ (Fig. 7a and **Suppl. Tables 1 and 2**). Three subjects with moderate symptomatic infection all required hospitalization for their illness but did not require mechanical ventilation and survived to hospital discharge. Three subjects with severe infection all required intubation and mechanical ventilation due to respiratory failure caused by COVID-19, and one of the three ultimately died of their illness. All subjects were experiencing their initial exposure to the SARS-CoV-2 spike antigen, either via the two dose BNT162b2 vaccine or via natural infection. We magnetically enriched CD3^+^ T cells from PBMC of the infected individuals to > 97% purity and proceeded to perform single cell RNA-seq with TCR sequencing (Fig. 7a).

We visualized all spike-specific CD4^+^ T cells in the experiment with a UMAP projection and found nine distinct clusters of antigen-specific CD4^+^ T cells (Fig. 7b). All nine clusters were found in both infected and vaccinated individuals, albeit with significantly different proportions of some clusters between the two groups when comparing the matched late time points (Fig. 7c). Relatively small differences existed between individuals with moderate and severe COVID-19 at the acute time point (**Suppl. Figure 5**).

Manual classification of the nine clusters based upon top differential gene expression revealed three clusters of central memory and two clusters of effector memory T cells (Fig. 7b and 7d). We observed separate populations of central and effector memory CD4^+^ T cells distinguished by high expression of the SLC2A3 glucose transporter gene that we term GLUT3^+^ central (1) and effector (3) memory populations. We also observed a population of anti-apoptotic central memory CD4^+^ T cells characterized by high expression of *BCL2* and *GIMAP5*^42^. These anti-apoptotic central memory CD4^+^ T cells composed a significantly higher proportion of antigen-specific CD4^+^ T cells in vaccinated individuals when compared to infected individuals (Fig. 7c). The proportions of central memory, effector memory and GLUT3^+^ effector memory CD4^+^ T cells were also significantly different at the late time points depending upon vaccination or infection as the initial antigen exposure route (Fig. 7c).

We found two populations of cytotoxic effector memory CD4^+^ T cells (5 and 8) characterized by high expression of cytotoxic cytokines (RANTES and MIP-1beta) and granzymes (Granzyme A, Granzyme K, and Granzyme H). We also observed a population of *FOXP3*- and *CTLA4*-expressing circulating regulatory T cells (6) and a population of regulatory CD52^high^ CD4^+^ T cells (7) that have previously been shown to suppress antigen-specific T cell responses via soluble CD52 ligation of Siglec-10 on target cells^43^. Foxp3^+^ regulatory T cells composed similar proportions of both infected and vaccinated antigen-specific CD4^+^ T cells, however, regulatory CD52^high^ CD4^+^ T cells were found in significantly higher proportions in vaccinated individuals (Fig. 7c).

We found that expanded clonotypes (Fig. 7e) of antigen-specific TCR with highly related TCR suggestive of clonal groups were noted in eight of the nine clusters and were found in every individual subject. Most of these clonal groups were found in the central memory clusters, clusters 0, 1 and 4. Only the relatively small MIP-1beta^+^ cytotoxic effector cluster (8) did not exhibit expanded clonal groups, likely due to the small size of this population.

The preponderance of central memory CD4^+^ T cells (clusters 0 and 1) in infected individuals compared to vaccinated individuals was accentuated in antigen-specific cells from the acute time point post-infection (Fig. 7f) where we discovered even higher proportions of central memory cells. Cytotoxic effector cells expanded to encompass a higher proportion of circulating antigen-specific CD4^+^ T cells between early and late sample time points in the infected individuals (Fig. 7f). The proportion of antigen-specific CD4^+^ T cells with effector memory (clusters 2 and 3), anti-apoptotic central memory (cluster 4) and regulatory (clusters 6 and 7) phenotypes remained stable between early and late time points in infected individuals (Fig. 7f).

## Discussion

Evaluating ongoing antigen-specific immune responses in the dLN of living humans has only recently been routinely accomplished with serial fine needle aspiration after vaccination^3,12,25–27,44^. In the present study, we evaluated antigen-specific CD4^+^ T cell responses to the SARS-CoV-2 spike antigen at the single-cell level in both the blood and the dLN. Using previously-published spike-specific TCR sequences and Trex as a tool to perform additional reverse epitope discovery^36,45,46^, we longitudinally tracked the evolution of large numbers of antigen-specific CD4^+^ T cells over time after SARS-CoV-2 mRNA vaccination in humans. This enabled detailed study of the human T_FH_ response at the total CD4^+^ T cell level, the antigen-specific CD4^+^ T cell level, and the individual CD4^+^ T cell clonotype level. The present work revealed three key findings: 1) total and antigen-specific T_FH_ in the human dLN after intramuscular vaccination exhibit distinct phenotypic profiles that vary significantly over time during the ongoing human GC response, 2) antigen-specific CD4^+^ T cells recognizing the same antigen and even the same epitope exhibit distinct phenotypes and clonotypic segregation between the blood and dLN during an ongoing GC response, and 3) antigen-specific CD4^+^ T cells in blood exhibit larger anti-apoptotic central memory and CD52^+^ regulatory populations following initial exposure to a new antigen via mRNA vaccination compared to infection.

Over the past decade, a growing number of supervised and unsupervised informatic tools have enabled the analysis of TCRs and antigen specificity. These approaches include amino acid motif-based quantifications^45^, edit distance-based clustering^47,48^, and neural-network-based architectures^49–51^. Using the latter, we created Trex, a TCR analysis platform built to combine deep variational autoencoders with gene expression data at the single-cell level. Although several methods have been previously published on the combination of TCR data and gene expression^36,50^, Trex differs in two key ways: 1) Trex offers up to 8 variational autoencoding models, a form generative artificial intelligence to encode TCR amino acid sequences into latent dimensional space; and 2) the latent dimensional space of the TCRs can be used adaptively to filter, cluster, or as a layer input for multimodal dimensionality reduction. In future, use of this technique to combine single-cell RNA, protein, and chromatin accessibility quantification with the vectorized TCRs could allow for even more comprehensive analysis of antigen-specific immune responses.

Despite identifying 11 individual T_FH_ transcriptional phenotypes in the dLN following vaccination, the majority of known antigen-specific T_FH_ exhibited the classical GC T_FH_ and IL-10^+^ T_FH_ phenotypes. We identified the largest number of overlapping paired TCR clonotypes between these two populations throughout the ongoing GC response, suggesting a common origin of these two effector T_FH_ populations. This is despite significant transcriptional differences between the two subsets that implies very different functional roles. IL-10^+^ T_FH_ express the highest levels of *IL21*, whereas *IL4* expression was almost exclusively observed in classical GC T_FH_. This finding is reminiscent of the segregation of these important functional cytokines in time and space within the mouse GC following infection^52,53^.

We observed minimal overlap between paired human antigen-specific CD4^+^ TCR sequences found in the dLN and those found in matched blood samples. Circulating T_FH_ were initially described as circulating CD4^+^ T cells with surface phenotypes similar to T_FH_ and activity that promotes B cell maturation *in vitro*^54,55^. It has been demonstrated that blood sampled early after antigen encounter either via vaccination^3,17–19,56^ or infection^57^ contains antigen-specific CD4^+^ T cells that are similar to GC-present T_FH_, and the size of this population correlates with the magnitude of the antigen-specific B cell and antibody response. Previously reported TCR sequencing of matched blood and secondary lymphoid organ T cells in a steady state without a known ongoing antigen-specific GC reaction suggested a clonal relationship between circulating CXCR5^+^ CD4^+^ T cells and secondary lymphoid organ CD4^+^ T cell populations^14^. This previous TCR sequencing work focused on sequencing of the TRB chain only and did not include an analysis of paired TCRs in both tissue compartments.

Our work now includes both paired sequencing and tracking of temporally-matched blood and dLN samples during an ongoing GC response. We observed very limited overlap between paired clonal T cell populations found in the blood and those found in the dLN, despite substantial numbers of spike-specific cells in each compartment. Our findings are in line with a recent report by Poon and Caron et al.^58^ showing minimal clonal overlap between paired TCRs from CD4^+^ T cells but detectable overlap between CD8^+^ T cell populations in blood and lymph node compartments sampled from deceased organ donors. Indeed, more than half of the overlapping clonotypes we identified in both dLN and blood were contaminating CD8^+^ T cells that composed less than 3% of the input blood T cell population. The three overlapping spike-specific CD4^+^ TCR we found in one subject out of the 131,651 TCRs sequenced from three individuals were found early in the dLN and late in the blood. This opens the possibility that these clones may have traveled through the dLN at the peak of the systemic antigen-specific CD4^+^ T cell response on day 28 but were not retained within the active GC. Alternatively, they may have been included in the early GC and represent the first emergence of circulating memory T_FH_ from the dLN. More study is required to completely understand the clonal T cell dynamics of the initiation, maintenance, and termination of the GC response as well as the relationship between dLN T_FH_ and circulating antigen-specific populations. Nevertheless, our data suggest that overlap between temporally-matched blood and dLN CD4^+^ T cell clonotypes found during the ongoing human GC response is rare. The present dataset did not address what occurs at the conclusion of the dLN GC response nor what occurs with antigen-specific clonotypes in the steady state outside of a GC response, and it is intriguing to speculate that many of these clonal antigen-specific T_FH_ populations emerge from the dLN to patrol in both blood and secondary lymphoid tissues as memory T_FH_.

Our tracking and transcriptional phenotyping of large numbers of antigen-specific CD4^+^ T cells in the present work allowed us to gain significant insights into the execution of the antigen-specific T_FH_ response after mRNA vaccination. There is substantial transcriptional variation over time in antigen-specific T_FH_ (Fig. 4), with peak transcriptional activity occurring around day 60 - five to six weeks after the final mRNA vaccine dose. This included elevated transcription of genes involved in T-cell activation, interleukin signaling, and cytokine signaling. Uniquely, we found upregulation of genes associated with CXCR4 signaling near the end of the GC response at d201, raising the possibility that this pathway may play a role in termination of the human GC.

Between 6–30% of antigen-specific T_FH_ we identified in the dLN were clonally identical throughout the GC, showing that founder populations persist for the full six months of the human GC response. We also found proliferating T_FH_ at every tested time point in the dLN, and this population included antigen-specific cells even at d201. This finding supports a model whereby clonal antigen-specific T_FH_ populations maintain homeostasis by continuous proliferation throughout the ongoing GC response.

Finally, we compared the mRNA vaccine-induced populations of antigen-specific CD4^+^ T cells in the blood with antigen-specific CD4^+^ T cells obtained from a cohort of HLA-DPB1*04^+^ individuals following infection with SARS-CoV-2. We found antigen-specific CD4^+^ T cells from each of 9 distinct transcriptional profiles in both vaccinated and infected subjects 3–6 months after infection. However, a significantly higher proportion of antigen-specific central memory CD4^+^ T cells in the vaccinated individuals exhibited high Bcl-2 expression and transcriptionally appeared more resistant to apoptosis, and vaccinated individuals also had significantly higher proportions of effector memory CD4^+^ T cells that belonged to a unique transcriptional group characterized by upregulation of the Glucose Transporter 3 gene. We found almost all of the unique regulatory CD52^high^ antigen-specific CD4^+^ T cell population^43^, in mRNA vaccinated subjects. Therefore, unique long-term transcriptional profiles are induced in memory antigen-specific CD4^+^ T cells depending upon the context of initial antigen exposure, either mRNA vaccination or viral infection.

Our work does have limitations. The present study evaluated CD4^+^ T cell responses from six mRNA-vaccinated subjects and six COVID-19-infected subjects. While our results are reproducible across this cohort, their broad applicability across larger populations of individuals cannot be adjudicated at this time. Our focus on HLA-DPB1*04^+^ individuals - while necessary to obtain sufficient numbers of antigen-specific cells for the unique analyses we performed - may have introduced unrecognized bias into our results and further validation of our findings would be required to ensure these findings are applicable to individuals without this HLA allele.

In conclusion, human antigen-specific CD4^+^ T cells in the dLN exhibit multiple transcriptional phenotypes that change over time following mRNA vaccination. The largest number of these cells exhibit the classical GC T_FH_ and IL-10^+^ T_FH_ phenotypes during the ongoing GC reaction. Antigen-specific dLN CD4^+^ T cells are phenotypically and clonotypically unique from circulating blood CD4^+^ T cells during an ongoing human GC response. Finally, circulating mRNA vaccine-induced antigen-specific memory CD4^+^ T cells exhibit transcriptional profiles suggesting a more constrained and apoptosis-resistant phenotype compared with cells from infected subjects. Together, our findings provide a single-cell atlas of human antigen-specific CD4^+^ T cell responses following vaccination and infection and demonstrate unique properties of these antigen-specific cells in dLN and blood.

## Methods

### Human subjects

We included samples from two prospective observational human cohorts. Demographics and HLA-typing of all included subjects are reported in **Supplemental Tables 1** and **2**. In the first cohort, human subjects who received the primary two-dose BNT162b2 mRNA vaccine series were prospectively enrolled into an observational study, WU-368. The WU-368 study was approved by the Washington University in St. Louis Institutional Review Board (approval # 2020–12-081). Complete details of the study cohort have been previously published^3,26,27^. Informed consent was obtained from each subject. Draining dominant lateral axillary lymph nodes ipsilateral to the deltoid muscle mRNA vaccination site were localized with ultrasound and sampled at the indicated time points with multiple passes of 6 separate 25-gauge needles using real-time ultrasound guidance. Each needle was flushed with 3 mL of R10 (RPMI 1640 media containing L-glutamine supplemented with 10% FBS, 100 U/mL penicillin-streptomycin) followed by three 1-mL rinses with R10. Any contaminating RBC were lysed with ACK hypotonic lysis buffer, dLN FNA cells were washed twice with P2 (1x PBS supplemented with 2% FBS and 2 mM EDTA) and cells were then counted and cyropreserved in 90% FBS with 10% DMSO before storage in liquid nitrogen until analysis. Matched blood samples at the indicated time points were obtained into EDTA-anticoagulated tubes and prepared to PBMC using Ficoll density gradient centrifugation. Contaminating RBC were removed from PBMC via hypotonic lysis, PBMC were washed, counted and cryopreserved in 90% FBS / 10% DMSO and kept in liquid nitrogen until analysis.

Human subjects in the second study, WU-350, experienced their first exposure to SARS-CoV-2 spike via acute infection in the first wave of the COVID-19 pandemic during the spring and summer of 2020. Subjects with acute symptomatic viral respiratory illness evaluated at Barnes Jewish Hospital, Saint Louis Children’s Hospital, Christian Hospital or affiliated Barnes Jewish Hospital testing sites, all located in Saint Louis, Missouri, USA were enrolled into a prospective observational cohort study. All subjects included in the present manuscript tested positive for SARS-CoV-2 with a clinical PCR test. Full details of the cohort and inclusion criteria have been previously published^41^. The WU-350 study was approved by the Washington University in St. Louis Institutional Review Board (approval # 2020–03-085). Informed consent was obtained from each subject or their legally authorized representative. Blood at the indicated time-points post onset of viral respiratory illness symptoms was collected into EDTA anticoagulated tubes and prepared to PBMC using Ficoll density gradient centrifugation. Contaminating RBC were removed by hypotonic lysis, PBMC were washed, counted and cryopreserved in 90% FBS / 10% DMSO and kept in liquid nitrogen until analysis.

### HLA-typing

Vaccinated individuals were HLA-typed by nanopore sequencing^59^. Genomic DNA was purified using the AllPrep DNA/RNA kit (Qiagen). Target HLA genes were amplified by long-range PCR (NGS LR kit, One Lambda) and sequenced following the SQK-LSK109 protocol on the R10.3 MinION flow cells (Oxford Nanopore Technologies). High-resolution HLA typing was assigned using the Athlon2 program.

For HLA-typing of infected individuals we extracted DNA from PBMCs using Zymo Quick-DNA Plus kits for use in the AllTYpe NGS 11-Loci Amplification Kit (One Lambda, Lot 014). HLA libraries were sequenced at 150×150 bp (MiSeq, Illumina), and the data were analyzed with TypeStream Visual (v3.0; One Lambda).

### dLN single-cell RNA-seq library preparation and sequencing

dLN FNA samples were thawed, washed with P2 and resuspended in P2. Chromium Single Cell 5’ Gene Expression Dual Index libraries and Chromium Single Cell V(D)J Dual Index libraries (10x Genomics) were prepared according to the manufacturer’s instructions without modifications. Both gene expression and V(D)J libraries were sequenced on a Novaseq S4 (Illumina) instrument, targeting sequencing depth of 50,000 and 5,000 read pairs per cell, respectively.

### T cell enrichment of PBMC populations for single-cell RNA-seq

Frozen PBMC samples were thawed, washed once with R10, and then washed with P2. PBMC were counted on a Cellometer Auto 2000 (Nexcelom) and resuspended to a final concentration of approximately 10^8^ cells/mL in P2. Total untouched CD3^+^ or positively selected CD4^+^ T cells were enriched using either the EasySep Human T Cell Isolation Kit or the EasySep Human CD4 positive selection kit II, respectively, with the EasyEights magnet (STEMCELL Technologies) all per the manufacturer’s instructions. Following enrichment, T cell populations were washed with P2, re-counted and resuspended in PBS supplemented with 0.05% BSA. Chromium single cell 5’ v2 gene expression and Chromium single cell V(D)J libraries (10x Genomics) were prepared according to the manufacturer’s instructions without modifications. Gene expression and V(D)J libraries were sequenced on a Novaseq S4 (Illumina) instrument.

Remaining T cells were stained for flow cytometry to verify the T cell enrichment. Of the remaining cells, 10^6^ enriched T cells were added to a round-bottom 96-well plate and washed twice in P2. A master mix was added to the cells with the following reagents for 20 minutes at 4°C: CD3 APC Fire 810 (HIT3a, Biolegend); CD4 PerCP (OKT4, Biolegend, to avoid blocking from positive selection); CD8 BV421 (RPA-T8, Biolegend); CD16 BV570 (3G8, Biolegend; CD14 APC (M5E2, Biolegend); CD19 BV750 (HIB19, Biolegend); Zombie NIR (Biolegend) diluted in Brilliant Staining buffer (50μL per test, BD Horizon) and P2. Following staining, cells were washed three times in P2 and then fixed with 1% paraformaldehyde (Electron Microscopy Sciences) for 20 minutes at 4°C. Cells were washed once in P2, then resuspended in P2, and stored at 4°C until analysis within 24–48 hours. Flow cytometry samples were run on an Aurora spectral flow cytometer using SpectroFlo v.2.2 software (Cytek). Flow cytometry data were analyzed using FlowJo v.10 (Treestar).

### Single-cell RNAseq processing and analysis

Filtered outputs of 10x Cell Ranger count and V(D)J pipelines were imported into R (v4.1) using the Seurat (v4.1.0 ) R package^60^. Filtering was applied on a sequencing run basis to remove cells with less than 100 features, more than 2.5-fold the standard deviation of feature numbers, and greater than 15% mitochondrial gene percentage. Doublets were estimated using the scDblFinder (v1.6.0) R package^61^. Individual cells were annotated using ProjecTILs (v2.0.3) R package^62,63^ and SingleR (v1.6.1) R package^64^ using the DICE annotation data set^65^. Clonotypes were added to the integrated Seurat object using the scRepertoire (v1.7.0) R package^66^. T cells were isolated based on the assignment of CD4/CD8 T cell annotation from ProjecTIL and the presence of a productive clonotype. Overall T cell dimensional reduction utilized 2,000 variable genes with the TCR genes removed to prevent bias in the manifold by clonality. The harmony (v0.1.0) R package^67^ was used in integrating multiple sequencing runs and generate the UMAP (dimensions = 1:15, epochs = 500) and clusters (resolution = 0.8, dimensions = 1:15, algorithm = 3). T follicular UMAP embedding and clustering utilized dimensions = 1:20 and a resolution of 0.5. CD8^+^ T cell designations were based on the examination of the distribution of CD8 expression, and a cut-off was set for *CD8A* ≥ 0.4. Spike-specific cells from vaccinated and infected donor peripheral blood were integrated using the Harmony R package using the individual sequencing run as the variable and dimensions = 1:30 with calculating UMAP (dimensions = 1:25, epochs = 500) and cluster (resolution = 0.5 and algorithm = 3). Gene expression UMAP overlays utilized the Nebulosa (v1.6.0) R package^68^. Gene set enrichment analysis was performed using the escape (v1.4.2) R package^69^ with the UCell approach^70^ and the Hallmark, Kegg, and BioCarta gene set libraries from GSEA^71^. TCR rarefication and extrapolation was performed using the iNEXT (v3.0.0) R package^72^ using the abundance of combined TRA and TRB clonotypes by patient and tissue and default settings in terms of bootstraps, knots and Hill numbers. TCR clustering was performed using the scRepertoire package and the clusterTCR function with the normalized edit distance threshold set to 0.85.

### TCR sequencing analysis and visualization

Spike-specific clonotype annotations were assigned for both TRA and TRB and derived from previously published data^3,28^ and the VDJdb database^29^. TCR sequencing motifs were created with the msa (v1.28.0) R package set to protein alignment with the ClustalW algorithm and max iteration = 30. The resulting aligned sequences were converted into seginer format and plotted with ggseqlogo (v0.1) R package. Single clonotype representation for single-cell analysis was performed similarly to the previously described CoNGA^36^. For a given combined TRA and TRB, a single transcriptome was selected based on the minimal Euclidean distance across all cells in the individual clonotype. Vectors for the TRA and TRB were calculated using the TCR autoencoder Trex (v0.99.7) R package translating the CDR3 amino acid sequence into a matrix based on the Kidera factors^73^. For the resulting RNA principal components and embedded TCR values, the first 15 dimensions were selected and rescaled using the mutual nearest neighbor approach with k=100 with the mumosa (v1.4.0) R package. The resulting values were then subjected to the phate algorithm with default settings with the PhateR (v1.0.7) R package^74^. Clustering was performed by generating a k-nearest neighbor igraph with the bluster (v1.6.0) R package and clusters were calculated using the Leiden algorithm from the leidenAlg (v1.0.3) R package with a resolution = 0.7 and number of iterations = 5. Putative spike-specific TCRs were derived from clusters where previously identified spike-specific TCRs were present. In addition, the putative TCRs were selected for the presence of either an alpha or beta chain that appeared in 2 or more donors and had not been previously shown to bind the spike epitope. Related putative-spike specific clones were called by identifying TRA or TRB CDR3 sequences within Levenshtein distance of two and shared V genes.

### Development of Trex autoencoding models

TCR embedding utilized training variational autoencoders on TRA and TRB CDR3 amino acid sequences, taking the AF, KF, or both converted numeric matrices with 0 padding to set CDR3 length of 60. The matrices were transformed into a 1-dimensional array, and values normalized across all sequences. Values with no variation were transformed into 0s. Alternatively, a one-hot autoencoding approach was also trained by converting the amino acid sequence to a matrix based on the individual amino acid along the sequence. A stacked autoencoder approach was utilized, similar to the previously described method^50^ with a 128–64-30–64-128 neuron structure. The bottleneck layer consists of a 30 neuron/vector embedding. Each autoencoder model was trained using the keras (v2.4.0) R package across 288,043 unique CDR3 AA TRA and 453,111 unique CDR3 AA TRB sequences across 15 single-cell data sets and 4 curated TCR databases – McPAS-TCR^75^, VDJdb^76^, IEDB^77^, and PIRD^78^ resulting in 8 models: TRA-AF, TRA-KF, TRA-both, TRA-OHE, TRB-AF, TRB-KF, TRB-both, TRB-OHE. The models were trained using 80:20 data split and hyperparameters were selected based on minimal Kullback-Leibler divergence value with a batch size of 128, learning rate of 0.001, and optimization using root mean square propagation. The TCR models and corresponding R package to run the embeddings with a Seurat or Single-cell Experiment object is available at https://github.com/ncborcherding/Trex.

### Putative spike-specific TCR transductants

Putative spike-specific TRA and TRB variable regions were combined *in silico* with murine constant regions (murine TRAC and murine TRBC2) modified to include additional cysteine residues in place of serine at position 57 in murine TRBC2 and threonine at position 47 in murine TRAC. Use of murine constant regions prevents pairing with endogenous human TCR following retroviral transduction of primary human T cells and the additional cysteine residues enhance alpha/beta constant region binding affinity increasing chimeric human variable/mouse constant TCR surface expression. Constructs containing the modified TRA and TRB were separated by a T2A sequence and synthesized to include a NotI and EcoRI restriction site at the 5’ and 3’ ends of the region of interest, respectively (GenScript). Synthesized constructs from GenScript were double-digested with NotI and EcoRI and cloned into the pMP71 retroviral vector^37^ and ligation was confirmed via sequencing of the recombinant plasmid. Recombinant pMP71 was used to transfect the 293Vec-RD114 retroviral packaging cell line (provided by BioVec Pharma) with the TransIT-LT1 (Mirus Bio) transfection reagent using the manufacturer’s protocol and recommended conditions. Transfection media was removed after 24 hours, replaced with fresh media, and retrovirus containing supernatents were harvested 24 hours later. Retroviral supernatants were stored at −80°C until used.

Human CD4^+^ T cells were enriched from cryopreserved PBMC using the EasySep Human CD4 Positive Selection Kit II (STEMCELL Technologies). Isolated T cells were cultured in R10–500 (R10 supplemented with 500 U/mL recombinant human IL-2 [BioLegend]) at 37°C with 5% CO_2_ and activated with the Miltenyi Biotec human T Cell Activation/Expansion kit according to the manufacturer’s instructions. 2 days after activation/expansion, activated T cells were purified from dead cell debris and activation beads with a Ficoll gradient. Cells were washed in R10, resuspended at 2×10^6^ per mL in R10–500, and plated on 24-well flat-bottom tissue culture plates.

TCR RD114 retroviral supernatants were thawed, layered on top of a 20% sucrose (w/v) gradient, and centrifuged in a microcentrifuge at 20,000 × g at 4°C for 1 hour. The supernatant was discarded and residual volume including the retroviral pellets were incubated with ViroMag beads (OZ Biosciences) for 15 minutes at room temperature. Retrovirus/beads were then added to the activated T cells in the 24-well plate and the plate was briefly centrifuged at 1600 × g for 1 minute before being placed on a pre-warmed magnet (OZ Biosciences) and incubated at 37°C with 5% CO_2_ for 15 minutes. Transduced T cells were cultured for at least 1 week prior to analysis with changes of R10–500 media, as needed.

### Intracellular cytokine staining mapping of human TCR transductants

250,000 to 500,000 transduced CD4^+^ T cells, a portion of which were confirmed to express the recombinant chimeric TCR using a murine TCR beta chain-specific monoclonal antibody (BV510, clone H57–597, BioLegend), were co-cultured with 100,000 EBV-transformed B cells from the experimental subject who expressed the index paired putative spike-specific TCR in the presence of various mapping pools of SARS-CoV-2 spike overlapping 17-mer peptides (NR-52402, BEI Resources). Each peptide was incubated at a final concentration of 1 μg/mL. Separate unstimulated control wells with equivalent concentrations of DMSO to the final concentration of DMSO found in the peptide-stimulated condition were included. Positive control phorbol 12-myristate 13-acetate (PMA, InvivoGen) and Ionomycin (InvivoGen) were added to separate wells. Cells in all conditions were co-cultured in R10 media supplemented with co-stimulatory antibodies against CD28 and CD49d (BD Biosciences). Samples with the appropriate stimulus were incubated for 1.5 hours before the addition of Brefeldin A and monensin (both from BD Biosciences) and then incubated for an additional 12–16 hours. Surface staining was performed followed by fixation in 1% paraformaldehyde, permeabilization with washing buffer supplemented with 0.1% w/v saponin (Sigma) and intracellular staining using fluorescently labeled antibodies directed against cytokine antigens. We used the following antibodies: CD3 PE-Cy7 (clone UCHT1, BioLegend), CD4 APC-Cy7 (clone SK3, BioLegend), murine TCR beta chain BV510 (clone H57–597, BioLegend), CD69 BV711 (clone FN50, BioLegend), IFN-gamma PE (clone B27, BioLegend), TNF-alpha PerCP-Cy5.5 (clone MAb11, BioLegend) and IL-2 APC (clone 5344.111, BD Biosciences). The panel included Zombie NIR viability stain (BioLegend). All antibodies were used at pre-titrated optimal staining concentrations. In a separate experiment performed on unstimulated TCR2 transductants, we performed the surface stain portion of the panel following incubation with an S_167–180_ HLA-DPB1*04:01 PE-labeled tetramer reagent (Washington University in Saint Louis tetramer core facility) for 15 minutes to confirm ICS results. All samples were acquired on a Cytek Aurora spectral flow cytometer and unmixed files were analyzed using FlowJo software (version 10, BD Biosciences). Final analysis was gated on live CD4^+^ T cells positive for murine TCR beta chain.

### Statistics

Heatmaps of gene sets were derived from the intersection of significant enrichment comparison (Bonferroni-adjusted p-value < 0.05) by ANOVA and Kruskal-Wallis H test for multiple comparisons and T-test and Wilcoxon Rank Sum test for binarized comparisons. Differential gene expression utilized MAST^79^ using the donor as a latent variable and a pseudocount of 0.1. Cluster proportion comparisons between antigen-specific T cells used the scProportionTest (v0.0.0.9) R package with 1,000 permutations. Code for the entire analysis is available at **Error! Hyperlink reference not valid.**.
